# Museum DNA reveals a new, potentially extinct species of rinkhals (Serpentes: Elapidae: *Hemachatus*) from the Eastern Highlands of Zimbabwe

**DOI:** 10.1371/journal.pone.0291432

**Published:** 2023-09-27

**Authors:** Tom Major, Pia Renk, Jens Reissig, Johanna L. A. Paijmans, Ellie Morris, Michael Hofreiter, Axel Barlow, Donald G. Broadley, Wolfgang Wüster

**Affiliations:** 1 Molecular Ecology and Evolution at Bangor, School of Natural Sciences, Bangor University, Bangor, Wales, United Kingdom; 2 Institute for Biochemistry and Biology, University of Potsdam, Potsdam, Germany; 3 Ultimate Creatures, Kelvin, Sandton, South Africa; 4 Department of Zoology, University of Cambridge, Cambridge, United Kingdom; 5 Natural History Museum of Zimbabwe, Bulawayo, Zimbabwe; State Museum of Natural History, GERMANY

## Abstract

Genetic information plays a pivotal role in species recognition and delimitation, but rare or extinct animals can be difficult to obtain genetic samples from. While natural history wet collections have proven invaluable in the description of novel species, the use of these historical samples in genetic studies has been greatly impeded by DNA degradation, especially because of formalin-fixation prior to preservation. Here, we use recently developed museum genomics approaches to determine the status of an isolated population of the elapid snake genus *Hemachatus* from Zimbabwe. We used multiple digestion phases followed by single strand sequencing library construction and hybridisation capture to obtain *12S* and *16S* rDNA sequences from a poorly preserved tissue sample of this population. Phylogenetic and morphological analyses in an integrated taxonomic framework demonstrate that the Zimbabwean rinkhals population represents an old and highly distinct lineage, which we describe as a new species, *Hemachatus nyangensis* sp. nov. Our phylogenetic dating analysis is compatible with venom spitting having evolved in response to the threat posed by early hominins, although more data are required for a robust test of this hypothesis. This description demonstrates the power of museum genomics in revealing rare or even extinct species: *Hemachatus* from Zimbabwe are only known from a small area of the Eastern Highlands known for high endemism. No living specimens have been seen since the 1980s, most likely due to dramatic land-use changes in the Eastern Highlands, suggesting that the species could be extinct. In view of its recognition as a highly distinct lineage, urgent action is required to determine whether any populations survive, and to safeguard remaining habitat.

## Introduction

Natural history collections are vast repositories of biodiversity, archiving material from much of the planet’s biodiversity, including species which may now be rare, difficult to find, or even recently extinct [1]. These collections have formed the bedrock of species descriptions for over two centuries. However, historically, there has been a major impediment to the use of historical specimens in genetic studies [2]. Several taxonomic groups, including reptiles, amphibians, and fish, are stored in alcohol as wet collections. Over time, this preservation method degrades DNA through hydrolysis. Many specimens are further subjected to fixation in formalin prior to storage, which causes additional miscoding lesions and protein-DNA cross links that may render sequence information unobtainable using conventional means [3, 4]. This has meant that the millions of specimens stored in the wet collections of natural history museums have remained largely inaccessible to genetic studies. Fortunately, the fast-evolving field of ancient DNA offers an opportunity to geneticists working with these historical samples. Ancient DNA approaches apply specialised extraction and sequencing library preparation protocols, in some cases combined with target enrichment procedures [4, 5], to successfully obtain DNA sequences from heavily degraded samples exceeding a million years in age [6]. These same techniques can be co-opted for use in similarly-degraded samples from natural history collections [4, 7]. These methods for obtaining genetic sequences from ancient and historical DNA are thus emerging as useful tools to open up the vast repositories of biodiversity to genetic analyses, including species delimitation [8]. Here, we apply these techniques to investigate the status of a poorly known and possibly extinct population of the elapid snake genus *Hemachatus* from the Eastern Highlands of Zimbabwe.

Closely related to true cobras of the genus *Naja*, the African elapid snake genus *Hemachatus (*Fleming, 1822) [9] currently contains a single species, *Hemachatus haemachatus* (Bonnaterre, 1790) [10], known colloquially as the rinkhals [11, 12]. These snakes are famous for displaying a defensive hooding posture when threatened, and spitting venom towards aggressors [13]. They are unique among Afro-Eurasian elapids in being viviparous. The genus has a southern African distribution encompassing parts of the Republic of South Africa, Lesotho, Eswatini and eastern Zimbabwe [11, 14].

The population in Zimbabwe has only been known to science since around 1920. A small, banded, and hooding snake described as a cobra was first reported from Nyanga in Zimbabwe’s Eastern Highlands by a Mr J.W. Barnes, a forestry officer on what was then Rhodes Inyanga Estates [14]. However, the species evaded positive identification, with no complete specimens available for study until 1961, when they were identified as *H*. *haemachatus* by Broadley [14, 15]. While a number of further specimens were collected until the 1980s, the continued survival of *Hemachatus* in Zimbabwe is now in doubt as a result of habitat alterations: the species was last seen in the country in 1988, and more recent expeditions have failed to find evidence of its continued existence [16]. Our genetic sample was taken from an individual that was collected by a Mr G. Puttent at the Nyanga Trout Hatchery in 1982. The specimen was given to one of us (D.G. Broadley) at the Natural History Museum of Zimbabwe (NMZB—9503) and subsequently examined. The specimen had been preserved and handed to the museum in a jar filled with some form of alcohol and labelled as such. Whether or not the specimen was ever exposed to formalin is unknown to us.

The available evidence suggests that the Zimbabwean rinkhals population was—or maybe still is—restricted to the Eastern Highlands of Zimbabwe. The Eastern Highlands are a centre of endemism and biodiversity, containing nine endemic animal species including a crab [17], a gecko [18], and a skink [19]. The rinkhals population in Zimbabwe is separated from the nearest southern localities in northeastern Mpumalanga Province, South Africa, by approximately 700 km. This separation, in conjunction with the status of the Eastern Highlands as a centre of endemism, led us to the hypothesis that this population could potentially represent an unrecognised taxon, distinct from *H*. *haemachatus*. Its apparently small range and recent decline (against a background of a global assessment of *H*. *haemachatus* as Least Concern [12, 20]) highlights the need to establish its taxonomic status to allow an assessment of its conservation status and potential recovery action [21, 22].

Here, we adopt an integrative taxonomic approach to determining the status of the rinkhals in Zimbabwe. Based on its geographical isolation, we hypothesise that the Zimbabwean rinkhals is likely to represent a distinct evolutionary lineage. In accordance with Padial et al. [23], we then assess its status by testing for evidence of temporal lineage separation using mtDNA sequence divergence to identify the rinkhals in Zimbabwe as a candidate species. In view of the limitations of mtDNA-based inferences, we then test for congruent phenotypic variation through univariate morphometric analysis to establish the different lineages as Confirmed Candidate Species [23].

## Materials and methods

### Historical specimen DNA extraction and library preparation

A 50 mg piece of liver tissue was removed from the historical specimen (NMZB 9503) and used for DNA extraction and Illumina sequencing library preparation. The procedures followed those described in [4]⁠, and were carried out using appropriate measures to avoid cross-contamination (i.e. clean room facilities, use of negative controls). Initially, the historical sample was subjected to a non-destructive digestion in guanidinium thiocyanate buffer (“Guanidine treatment” [4]) followed by DNA purification using the method of [5]. The undigested tissue pellet was then re-digested using a Proteinase K buffer (“Proteinase K re-digestion treatment” [4]) and the same method of DNA purification applied. The purified DNA extracts were then quantified using a Qubit fluorometer with high sensitivity reagents. Dual indexed sequencing libraries were prepared from 12 ng of each DNA extract using a single stranded method [24] with the modifications described in [4]. Uracil DNA-Glycosylase and Endonuclease VIII were used to excise uracil residues, which can occur in historical DNA molecules as a result of cytosine deamination. The optimal number of indexing amplification cycles was determined in advance using qPCR analysis of the unamplified library [4]. Amplified libraries were quantified using a Qubit fluorometer with high sensitivity reagents and an Agilent TapeStation instrument with D1000 reagents. Shotgun sequencing was performed using an Illumina NextSeq 500 Instrument producing 75 bp single-end reads [25].

### Assessment of endogenous DNA content

We interrogated the shotgun data from the historical sample for the presence of rinkhals DNA. Data processing was carried out using the publicly available BEARCAVE scripts (https://github.com/nikolasbasler/BEARCAVE). Briefly, adapter sequences were trimmed using the software CutAdapt [26], requiring a single base overlap. Reads < 30 bp after trimming were discarded. The trimmed reads were then mapped to the phylogenetically closest reference nuclear genome sequence available, that of the king cobra (*Ophiophagus hannah*) [27] using the aln algorithm of the software bwa [28], with subsequent filtering for mapping quality (-Q 30) and removal of potential PCR duplicates (rmdup) using samtools [29]. We also mapped reads to the mitochondrial genome of the Chinese cobra (*Naja atra*; GenBank accession: EU913475), using a range of allowed mismatches in bwa (-n 0.04, 0.01, 0.001). None of these analyses were able to recover a substantial number of mapped reads, leading us to conclude that the proportion of endogenous rinkhals DNA molecules in DNA extracted from the historical sample was extremely low. We therefore chose to target the mitochondrial genome of the historical specimen for recovery using hybridisation capture.

### Draft assembly of a modern rinkhals mitochondrial genome

Hybridisation capture requires nucleotide “baits’’ with high sequence similarity to the target region(s). These can take the form of PCR products which can be converted into baits for hybridisation capture [30]. However, to our knowledge, no suitable primers exist for the amplification of the complete mitochondrial genome of the rinkhals or any close relatives. To overcome this, we extracted DNA from a ventral scale tissue sample from a modern rinkhals specimen and carried out shotgun sequencing. DNA was extracted using a commercial kit (Qiagen DNeasy) and a dual-indexed Illumina sequencing library prepared using the double-stranded method described in [31], with the modifications described in [32]. Sequencing and adapter trimming of the data were carried out as described above. We then generated a draft assembly of the mitochondrial genome sequence of the modern specimen using an iterative mapping method with the program MITObim [33], using the Chinese cobra mitochondrial genome as the initial bait sequence and default parameters. The resulting draft assembly was imperfect with several large sequence gaps. However, it was sufficient for the design of PCR primers to enable the amplification of the modern rinkhals mitochondrial genome using long-range PCR.

### Long-range PCR primer design, bait preparation, and hybridisation capture

From the draft modern rinkhals mitochondrial genome sequence, we designed two sets of primers to amplify the mitochondrial genome in two overlapping sections using Primer3Plus [34], selecting a preferred primer length of 18–22 bp and a maximum product size of 10 kb. Specificity of the designed primer sets were then checked by BLASTn analysis against the NCBI nucleotide database. Long-range PCR was carried out using these primers and the DNA extract of the modern rinkhals as template, following the procedure described in [30]. Hybridisation capture baits were then prepared by pooling the PCR products in an equimolar ratio, shearing using a Covaris S220 focused ultrasonicator, after which biotinylated adapters were ligated following the procedures described in [30].

Using these baits, we performed two rounds of hybridisation capture on the historical Zimbabwean rinkhals sample libraries, with library application cycles selected using qPCR analysis after each capture round [30]. We also prepared an amplicon sequencing library from the modern rinkhals sheared long-range PCR products using the double-stranded method described above [25]. Libraries were sequenced as described above.

### Reconstruction of the historical mitochondrial sequence

We used the amplicon data from the modern rinkhals to generate an improved rinkhals mitochondrial reference sequence for mapping. This was achieved using a custom iterative mapping procedure that involved mapping of the amplicon data to the Chinese cobra mitochondrial reference using the bwa mem algorithm, and the removal of unmapped reads and non-primary alignments using samtools. A consensus sequence was then called in Geneious v7 using a 50% majority rule, retaining the reference sequence for regions lacking mapped reads. This procedure was repeated ten times, each time using the newly generated consensus sequence as mapping reference, at which point no new reads were mapped. The consensus sequence generated in the final iteration used a 90% majority rule, required a minimum depth of 3 reads, and did not retain the reference sequence for regions lacking mapped reads. The original reads were then re-mapped using the more stringent bwa aln algorithm using this consensus as reference sequence, and a final consensus generated from this alignment in Geneious using an 85% majority rule and a minimum depth of 10 reads.

We used this improved rinkhals mitochondrial reference sequence to map both shotgun and hybridisation capture data from the historical specimen, using bwa aln with default mismatch parameter and the filtering methods described in “Assessment of endogenous DNA content”. The aligned data from the historical specimen was then viewed using samtools tview. Being a small and manageable mitogenome, the coverage was evaluated by eye to determine which regions of the mitogenome we could be most confident in. Two regions with a coverage of three or more reads were found which roughly corresponded to regions of the *12S* ribosomal RNA gene (563 bp) and *16S* ribosomal RNA gene (621 bp).

### mtDNA sequencing of additional modern specimens

**Fig 1:**
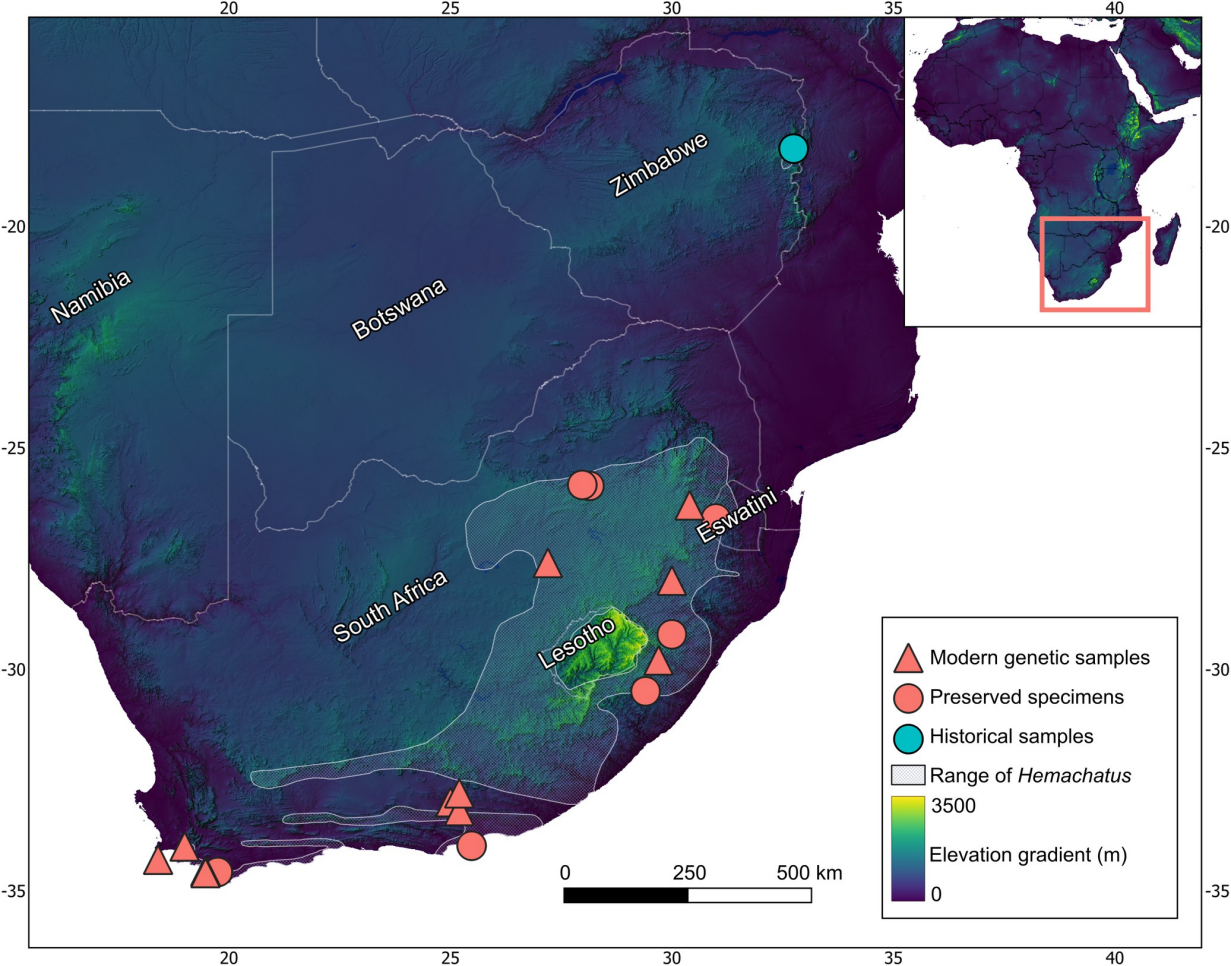
Localities for historical and modern rinkhals included in the analyses. Ten individuals from South Africa included in the morphological analysis were excluded from the map as their locality was only recorded to province level (S4 Table in S1 File). Preserved specimens from South Africa are those used in the morphological analysis. Overlaid locations have been adjusted to improve visibility. Public domain elevation data were sourced from the USGS Earth Resources Observatory and Science Center [46]. Range layer for Hemachatus reprinted from [20] under a CC BY license, with permission from the IUCN, original copyright 2022.

We collected seven tissue samples of modern rinkhals (*H*. *haemachatus*) from across its range in southern Africa (Fig 1). Samples were collected under Cape Nature permit no. AAA004-00127-0035 or from captive specimens of known provenance, and this work took place with approval from the Bangor University Animal Welfare and Ethics Review Board. DNA was extracted as described for the modern sample above and quantified using a Nanodrop spectrometer. We then PCR amplified and Sanger sequenced the mitochondrial *12s* and *16s* genes, overlapping the recovered regions for our historical specimen. Primers and relevant target genes are presented in Table 1. PCR was carried out in 15 μl volumes using GoTaq® Green Master Mix and 1–3 μl of DNA template. Thermocycling programmes are provided in S1 and S2 Tables in S1 File. Following PCR, each sample was prepared for sequencing with the addition of 1 μl exonuclease 1 (20 U/μl) and 2 μl of thermostable shrimp alkaline phosphatase (1 U/μl), followed by a 15 minute incubation at 37°C and an enzyme inactivation period of 15 minutes at 85°C. The *12S* fragments were sequenced in both directions and we created a consensus of the two sequences using Geneious version 2020.1 [35].

**Table 1:** Primers used in the PCR process.

Genes	Primers (’5 to 3’)	Reference
*12S*	*12S* rRNA, AAAGTATAGCACTGAAA	[36]
	tRNA-Val, GTCGTGTGCTTTAGTCT	[36]
*16S*	L2510, CGCCTGTTTATCAAAAACAT	[37]
	H3059, CCGGTCTGAACTCAGATCACGT	[38]

### Phylogenetic analysis and molecular dating

To investigate the timing of the divergence between Zimbabwean and southern African populations of *Hemachatus*, we aligned our *16S* and *12S* sequences with corresponding sequences from other species of the genera *Naja*, *Pseudohaje*, *Walterinnesia*, *Aspidelaps* and, as outgroup, *Ophiophagus*, taken from GenBank and our own unpublished data. To increase the overall robustness of the tree, we concatenated this alignment with additional sequences of the mitochondrial genes for cytochrome *b* (cyt-*b*) and NADH dehydrogenase subunit 4 (ND4) from [13] for all taxa except the Zimbabwe *Hemachatus*. All sequences were aligned in MEGA 11 [39] using the MUSCLE alignment algorithm and default settings [40]. In both *12S* and *16S*, a short region of hypervariable sequence was excluded from further analysis.

Phylogenetic reconstruction and molecular dating were carried out in BEAST v. 1.10.4 [41]. To calibrate the tree, we constrained several highly supported key nodes according to the multilocus phylogeny of Kazandjian et al. [13], and constrained and applied normal priors to their date. The mean date obtained by the previous molecular dating study [13] was set as the mean, with a standard deviation of 0.01, effectively fixing the node date. The constrained nodes and their dating were the cobra clade *Aspidelaps* + *Walterinnesia* + *Hemachatus* + *Pseudohaje* + *Naja* (19.7 Mya), *Hemachatus* + *Naja* + *Pseudohaje* (17.0 Mya), and the subgenera *Afronaja* (6.74 Mya), *Naja* (5.44 Mya) and *Uraeus* (3.71 Mya). A lognormal uncorrelated relaxed clock model was used. The optimum data partitioning scheme was identified using Partitionfinder v. 1.1.1 [42], using the greedy search algorithm in conjunction with PhyML [43]. The identified partitions were *12S*, *16S*, and the three codon positions of the combined, concatenated protein-coding genes, cytochrome b (*cyt-b*) and NADH dehydrogenase 4 (*ND4*). We implemented the general time-reversible model with four gamma categories, empirical base frequencies, gamma shape parameter and proportion of invariable sites for each partition, as recommended by Partitionfinder, with the departure that, for operational reasons, we used the GTR+I+G model for the third codon positions of the two protein-coding genes, as opposed to GTR+G, as supported by Partitionfinder. Since most nodes of the phylogeny were interspecific, we implemented a Yule Speciation Process Tree. The analysis was run for 20 million generations with a 10% burn-in period. Burn-in and the effective sample size (ESS) of all parameters were verified using Tracer.

### Morphological analysis

We included 22 individuals of *Hemachatus* from South Africa and 14 from Zimbabwe in the morphological analysis (S4 Table in S1 File). We recorded sex, snout-vent length (SVL), and tail length (TL). We counted the number of nape scale rows, midbody scale rows, pre-cloacal scale rows, cloacal scales, subcaudal scales, upper labials, upper labials entering orbit, lower labials, lower labials in contact with the anterior sublinguals, preocular, and postocular scales. Due to the state of preservation of the specimens, data were not recorded for every character in all snakes (Table 1, S4 Table in S1 File). Depending on the character, we used chi-squared, Fisher’s exact, and two-way analysis of variance (ANOVA) tests to evaluate the influence of group and sex on all characters. As size can be a confounding factor in the analysis of growth-related characters, and because TL is likely to be sexually dimorphic, we performed a two-way analysis of covariance (ANCOVA) on TL data using SVL as the covariate [44].

Finally, since even long-separated lineages may lack absolutely diagnostic characters, we conducted a principal component analysis (PCA) using the meristic variables that differed significantly between the two populations in order to visualise the distinctness of the two forms in multidimensional morphospace free of the confounding effects of a priori group assignment. This analysis included midbody scale rows, nape scale rows, number of ventral scales, and number of subcaudal scales. All characters were standardised to zero mean and unit standard deviation prior to analysis. For the PCA, we performed a singular value decomposition of the covariance on data from 12 snakes from South Africa and 11 from Zimbabwe.

### Nomenclatural acts

The electronic edition of this article conforms to the requirements of the amended International Code of Zoological Nomenclature, and hence the new names contained herein are available under that Code from the electronic edition of this article. This published work and the nomenclatural acts it contains have been registered in ZooBank, the online registration system for the ICZN. The ZooBank LSIDs (Life Science Identifiers) can be resolved and the associated information viewed through any standard web browser by appending the LSID to the prefix “http://zoobank.org/”. The LSID for this publication is: urn:lsid:zoobank.org:pub:CB9F0F49-FCDA-4288-A693-5187A810B24C. The electronic edition of this work was published in a journal with an ISSN, and has been archived and is available from the following digital repository: LOCKSS.

## Results

### Genetic analysis

We recovered 14,791 bp of unambiguous sequence for the mitogenome of the modern rinkhals sample ZRP2264. The missing parts of the mitogenome corresponded primarily to the two control regions (many snakes have two identical or near-identical mitochondrial control regions [45]). Against this reference, we were able to align 349 bp of *16S* and 394 bp of *12S* unambiguous sequence of the Zimbabwean sample.

For phylogenetic and molecular dating analyses, we aligned a total of 2,311 base pairs of mitochondrial DNA (*12S*: 512 b.p.; *16S*: 483 b.p.; *cyt-b*: 657 b.p.; *ND4*: 659 b.p.) for all specimens except the Zimbabwean rinkhals, for which only partial *16S* and *12S* sequences were available. The sequences used and their GenBank accession numbers are shown in S3 Table in S1 File.

**Fig 2:**
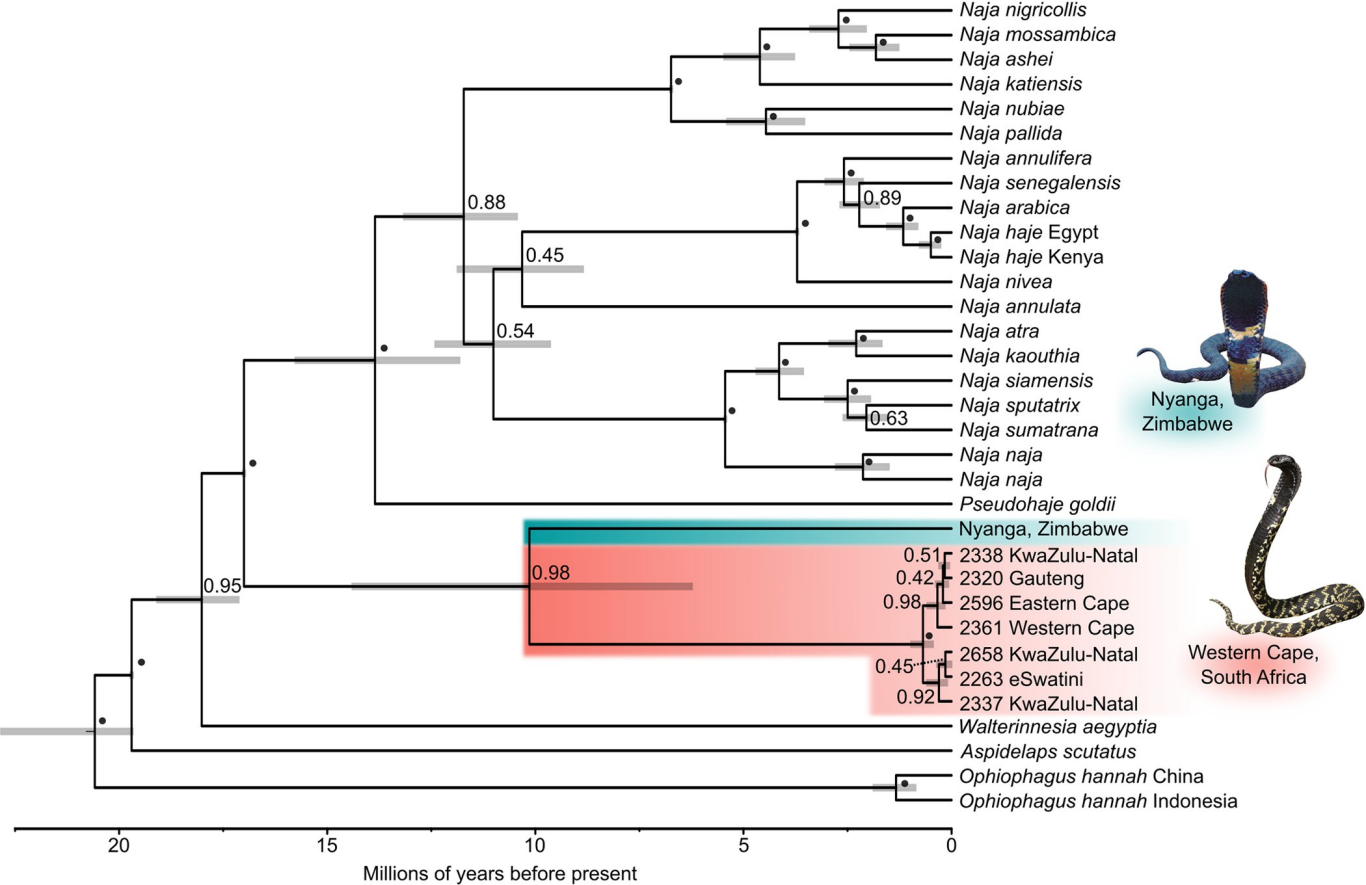
Dated Bayesian phylogenetic tree of the cobra group of elapids. Grey bars denote 95% HPD confidence intervals for divergence times; nodes lacking grey error bars were used as calibration points. Node labels represent Bayesian posterior probabilities (BPP), with black dots denoting BPP ≥ 0.95. The clade representing *Hemachatus* individuals from South Africa and Eswatini is highlighted in salmon pink, and the divergent Zimbabwe lineage in blue-green.

All parameters of the analysis were confirmed to have reached convergence in Tracer, with ESS values of over 500 in all cases. The maximum clade credibility tree (Fig 2) confirms the monophyly of *Hemachatus* with high levels of support (posterior = 0.98), and an estimated age of 10.14 Mya (95% HPD = 6.25–14.39 Mya) for the split between the Zimbabwean *Hemachatus* and the samples from the remainder of the range. Excluding the Zimbabwe lineage, the age of first divergence within *H*. *haemachatus* is 0.69 Mya (95% HPD = 0.46–0.96 Mya).

### Morphology

**Fig 3:**
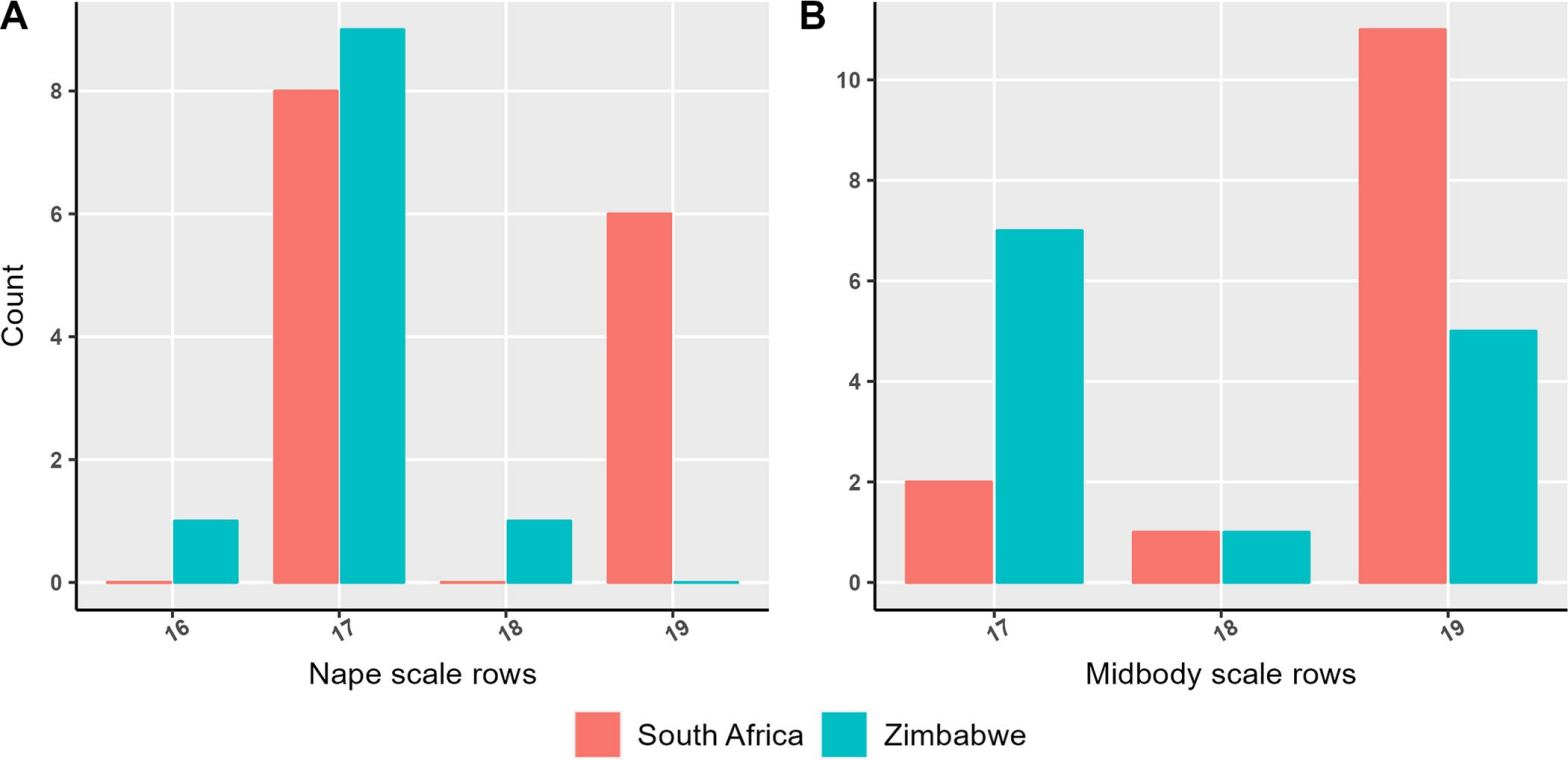
Bar plots showing the number of dorsal scale rows snakes possessed at the nape (A) and at midbody (B).

**Fig 4:**
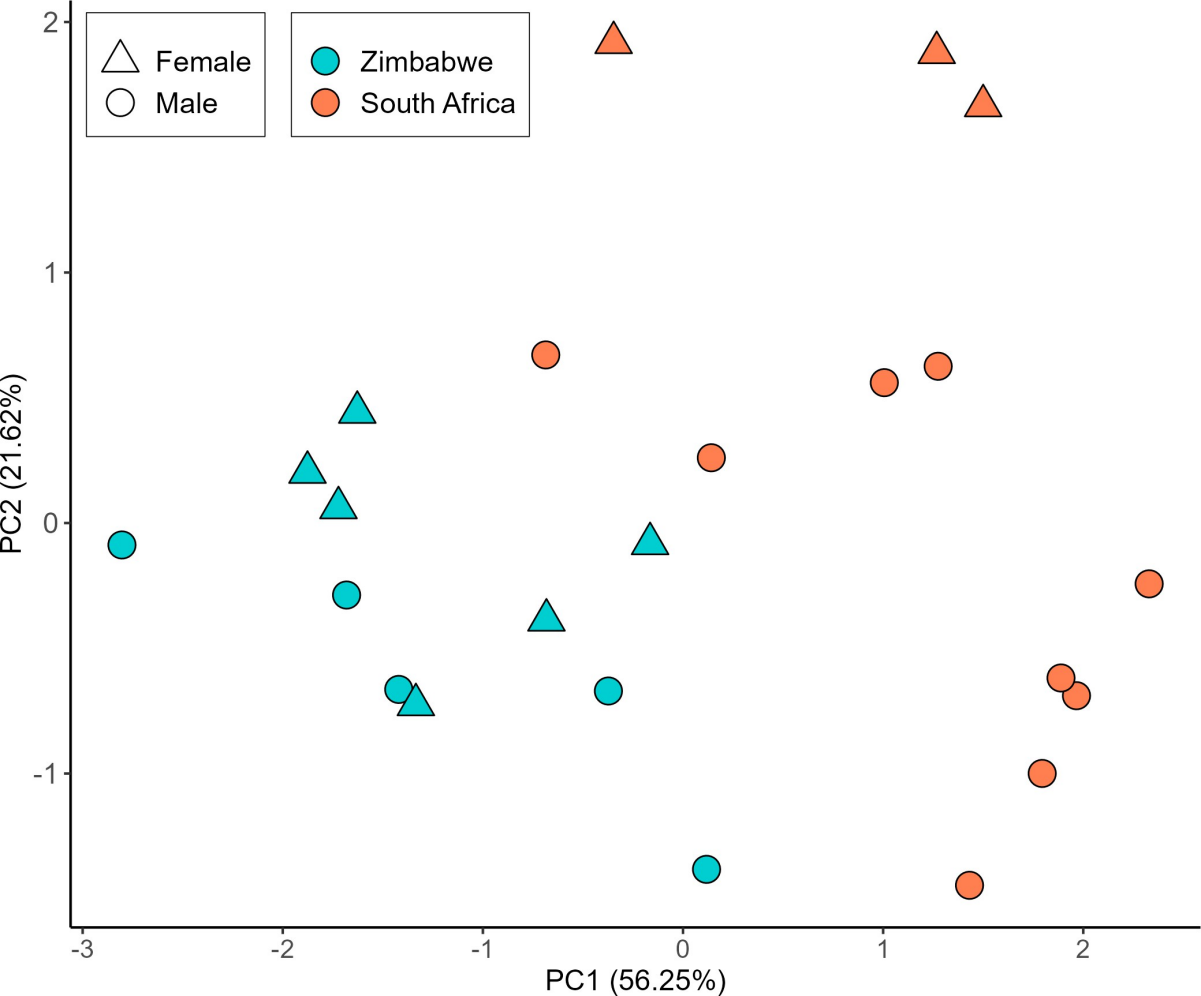
Ordination of South African and Zimbabwean specimens of *Hemachatus* along the first two principal components of the PCA of morphological data. Variables included are midbody scale rows, nape scale rows, number of ventral scales, and number of subcaudal scales. The first and second principal components represent 56.25% and 21.62% of the total variance in the data, respectively.

Our univariate morphological analyses demonstrate that rinkhals from Zimbabwe possess significantly fewer midbody scale rows, nape scale rows, ventral scales, and subcaudal scales than those from South Africa (Tables 2 and 3, and Fig 3). Our PCA visualises the differences in overall phenotype across the four characters included, despite the absence of absolutely diagnostic single characters. Eigenvector coefficients of the characters in the PCA are presented in Table 4: broadly, higher counts in all meristic characters, especially subcaudals, are associated with higher PC 1 scores, and higher ventral scale counts with high PC 2 scores. Consistent with the univariate analyses, South African specimens exhibit generally higher PC 1 scores than those from Zimbabwe (Fig 4), reflecting their generally higher scale counts, especially subcaudals.

**Table 2:** Results of statistical tests comparing the morphological characters of South African and Zimbabwean *Hemachatus*. ANOVA, chi-squared tests, and Fisher’s exact tests were used where appropriate. In the *P* column, asterisks highlight significant differences or interactions (*P* < 0.001 ***, < 0.01 **, < 0.05 *).

Character	Test	Effect	d.f.	Value	*P*
Midbody scale rows (17–18, or 19)	Chi-squared test	population	1	*X*^*2*^ = 4.49	0.034 *
Nape scale rows (16–18, or 19)	Fisher’s exact test	population	-	-	0.02 *
Pre-cloacal scale rows (11 or 13 or 14)	Fisher’s exact test	population	-	-	0.697
Supralabials (6 or 6.5 or 7)	Fisher’s exact test	population	-	-	1.000
Subcaudals	Two-way ANOVA	population	1	F = 25.839	< 0.001***
Subcaudals	Two-way ANOVA	sex	1	F = 4.009	0.055
Subcaudals	Two-way ANOVA	population * sex	1	F = 0.133	0.718
Ventrals	Two-way ANOVA	population	1	F = 9.036	0.006 **
Ventrals	Two-way ANOVA	sex	1	F = 16.935	< 0.001 ***
Ventrals	Two-way ANOVA	population * sex	1	F = 1.822	0.189
Tail length	Two-way ANCOVA	population	1	F = 2.659	0.125
Tail length	Two-way ANCOVA	sex	1	F = 14.547	0.002 *
Tail length	Two-way ANCOVA	population * sex	1	F = 1.910	0.189

**Table 3:** Comparison of standard scale counts between *Hemachatus* from South Africa and Zimbabwe. Shown are the mean and standard deviation, with range in brackets, and sample size. Sexually dimorphic characters are divided by sex.

Character	South Africa	Zimbabwe
Midbody scale rows	18.6 ± 0.72 (17–19), *N = 14*	17.8 ± 0.95 (17–19), *N = 13*
Nape scale rows	17.9 ± 0.99 (17–19), *N = 14*	17 ± 0.43 (16–18), *N = 11*
Subcaudal scales females	39 ± 2.6 (35–40), *N = 6*	33.8 ± 2.3 (30–37), *N = 6*
Subcaudal scales males	40.9 ± 3.1 (35–46), *N = 14*	36.3 ± 1.8 (34–38), *N = 5*
Ventral scales females	141 ± 6.5 (129–148), *N = 5*	128 ± 1.6 (126–130), *N = 5*
Ventral scales males	129 ± 6.9 (117–138), *N = 13*	122 ± 1.7 (119–124), *N = 5*

**Table 4:** Eigenvector coefficients for each scaled variable from the principal component analysis, eigenvalues, and the total variance explained by each principal component. The variables that contributed most to each principal component are in bold.

	PC1	PC2
Subcaudals	**0.576**	0.140
Ventrals	0.427	**0.774**
Nape rows	0.490	-0.545
Midbody rows	0.495	-0.291
Eigenvalue	2.245	0.865
Percentage of total variance	56.25	21.62

### Species delimitation and systematics

The *Hemachatus* population in Zimbabwe is separated from the main range of *H*. *haemachatus* by over 700 km, and we used this separation to hypothesise that it may constitute a separate lineage (Fig 1). We tested for evidence of temporal lineage separation and found deep mtDNA sequence divergence between the Zimbabwe population and rinkhals from South Africa and Eswatini. Finally, phenotypic differentiation was confirmed using univariate analyses of standard morphological characters (Table 2). These lines of congruent evidence indicate that our candidate species, the *Hemachatus* population from Nyanga, Zimbabwe, represents an ancient lineage distinct from *H*. *haemachatus* in Lesotho, South Africa, and Eswatini (Figs 1 and 2), and therefore a separate species. As there are no published, available names for the Zimbabwean rinkhals, we here describe it as a new species.

#### *Hemachatus nyangensis* sp. Nov

Reissig, Major, Renk, Barlow, Paijmans, Morris, Hofreiter, Broadley, and Wüster.

Suggested common name: Nyanga rinkhals.

urn:lsid:zoobank.org:act:7BFD5FC9-556F-4C59-9246-83585E32E56D

### Holotype

**Fig 5:**
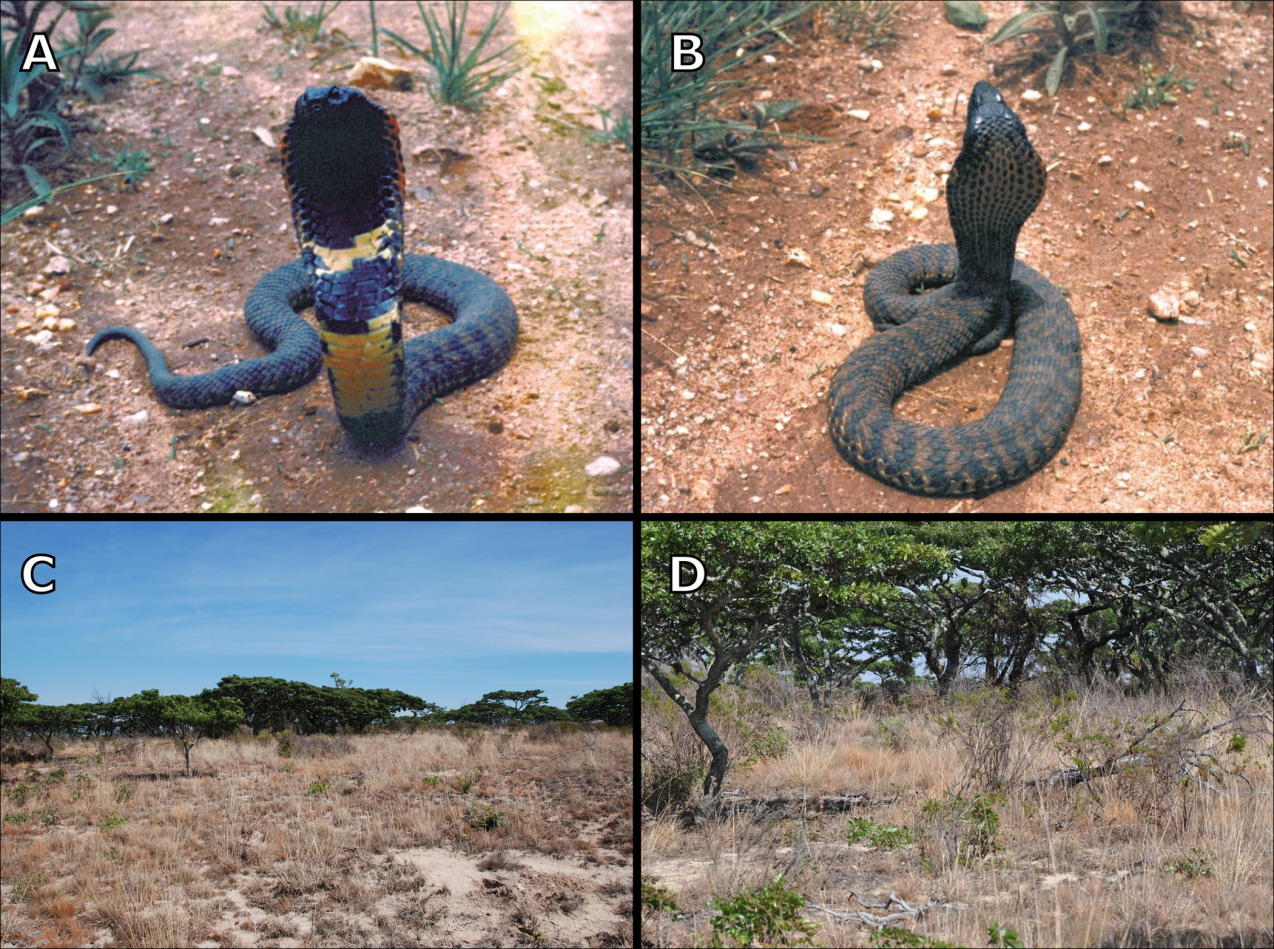
(A and B) *Hemachatus nyangensis* sp. nov. specimen in life, displaying defensive hooding posture. (C and D) Miombo woodland and grassland habitat of *H*. *nyangensis* sp. nov.

**Fig 6:**
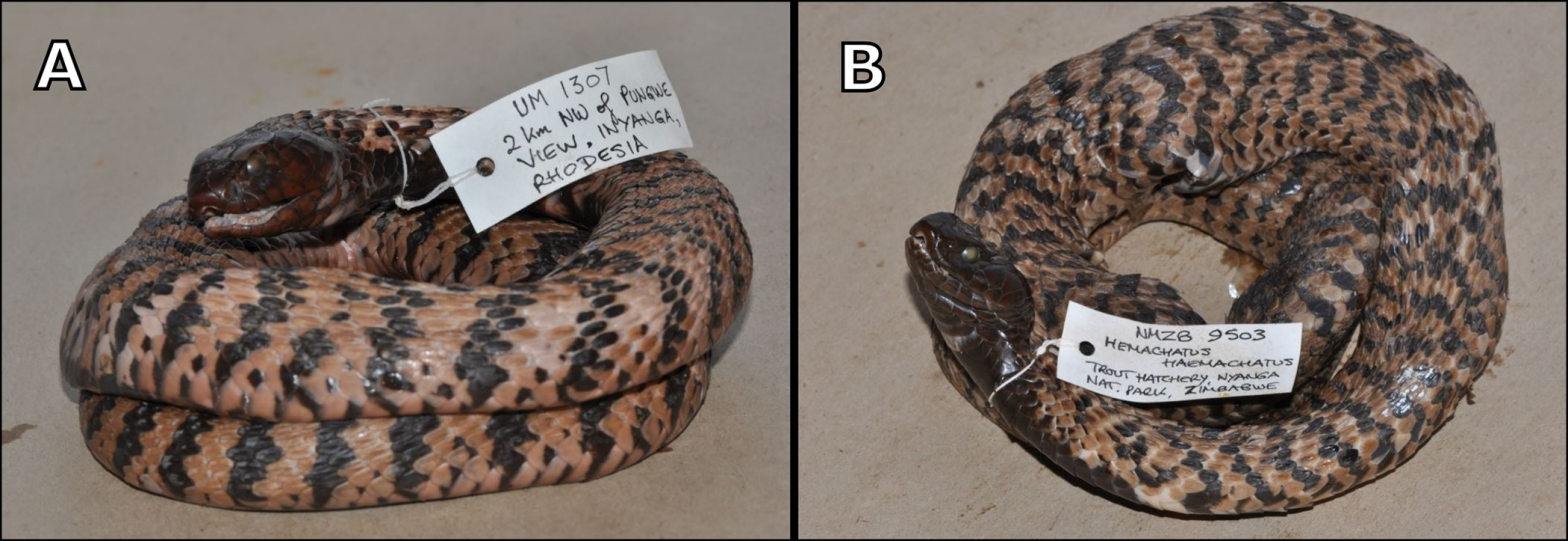
(A) Male holotype specimen of *Hemachatus nyangensis* sp. nov. from Nyanga National Park, following preservation (NMZB—UM 1307). (B) Female paratype specimen of *Hemachatus nyangensis* sp. nov. from Nyanga National Park, following preservation (NMZB 9503).

NMZB—UM 1307, a male specimen (Figs 5 and 6) collected from 2 kilometres northwest of Pungwe View, Nyanga National Park, Nyanga District, Manicaland Province, Republic of Zimbabwe, 18.42°S, 32.78°E, elevation 1122 m, by D. G. Broadley on 23/11/1961. This individual was killed by a car strike.

Dimensions: A male specimen, snout-vent length 635 mm, tail length 133 mm, total length 768 mm.

Body scalation: 18 scale rows around hood, 19 around midbody, 13 one head length ahead of the vent, all strongly keeled and oblique. Dorsal scales strongly keeled, oblique. Vertebral row not enlarged. 123 ventrals, 38 subcaudals, all divided except for first 4 which are single, anal single.

Head scalation: 7/7 supralabials, 3^rd^ & 4^th^ contact orbit, 5^th^ largest; 8/8 infralabials, first 4 contact anterior chin shields; 1/1 preocular; 3/3 postoculars; 2/2 anterior temporals; 2/3 posterior temporals; rostral almost as deep as broad, clearly visible from above.

Pattern: Head black, body orange-brown with forty-three narrow black cross-bands, equal in width to the light interspaces, extending from just behind the neck till just behind the cloaca; chin dark brown, throat black with two white cross-bands, rest of belly and tail grey.

### Paratype

NMZB 9503, a female specimen (Fig 6) collected on the grounds of the Nyanga Trout Hatchery, Nyanga National Park, Nyanga District, Manicaland Province, Republic of Zimbabwe, by G. Puttent in 1982. Cause of death is unknown but may have been killed by a person or struck by a vehicle.

Dimensions: A female specimen, snout-vent length 625mm, tail length 121mm, total length 746mm.

Body scalation: 17 scale rows around hood, 17 around midbody, 13 one head length ahead of the vent, all strongly keeled and oblique. Dorsal scales strongly keeled, oblique. Vertebral row not enlarged. 130 ventrals, 34 subcaudals, all divided, anal single.

Head scalation: 7/7 supralabials, 3^rd^ & 4^th^ contact orbit, 5^th^ largest; 8/8 infralabials, first 4 contact anterior chin shields; 1/1 preocular; 3/3 postoculars; 2/2 anterior temporals; 3/3 posterior temporals; rostral almost as deep as broad, clearly visible from above.

Pattern: Head black, body yellow-brown with narrow black cross-bands, less distinct than in NMZB—UM 1307, and approximately equal in width to the light interspaces; chin dark brown, throat black with white cross-bands, rest of belly and tail grey.

Note: this is the specimen from which the DNA sequences used in this study were obtained.

### Etymology

The specific epithet *nyangensis* means “from Nyanga” in Latin and is chosen to reflect the distribution of the species in the Nyanga district of Zimbabwe, the only area in which it has been documented.

### Diagnosis

Distinguishable from its relative *Hemachatus haemachatus*, for which we propose the common name “Southern Rinkhals” and which occurs in South Africa, Lesotho, and Eswatini, by its isolated distribution in eastern Zimbabwe. Morphologically, *Haemachatus nyangensis* sp. nov. generally has overall lower body scale counts than its southern relative: it usually has fewer nape scale rows (16–18 instead of 17–19), midbody scale rows (commonly 17–19 vs usually 19) (Fig 3), fewer subcaudal scales in both females (30–37 vs 35–40 in *H*. *haemachatus*) and males (34–38 vs 35–46) and generally fewer ventral scales in both females (126–130 vs 129–148) and males (119–124 vs 117–138) (Table 3). The new species is genetically diagnosable through differences in the *12S* and *16S* mitochondrial sequence. The description of this species means that the genus *Hemachatus* is no longer monotypic.

### Variation

Dorsal scale rows on neck 16–18, at midbody 17–19, before vent 11–13; ventrals 119–130, subcaudals 30–38, all divided (up to the first six rows of subcaudals can be singular). Males have lower ventral (119–124) and higher subcaudal (34–38) counts, whilst females have higher ventral (126–130) and lower subcaudal (30–37) counts. Supralabials 7 (rarely 6), the third and fourth (rarely third only) in contact with the orbit; infralabials 8 (rarely 7), the first 4 (rarely 3) in contact with the anterior chin shields; preocular 1; postoculars 3; temporals 2+2 or 2+3. The head is black, body yellow-brown or orange-brown, sometimes even reddish, with narrow black cross-bands, equal in width to the light interspaces. The black cross-bands can give the animal a clean appearance or sometimes even make it appear mottled. Some uniformly grey specimens have been recorded; chin dark brown, throat black, usually with two distinct white cross-bands, rest of belly and tail grey.

### Largest recorded

865+150 = 1015 mm, from Nyanga National Park, Zimbabwe (NMZB–UM 16098).

### Remarks

This new taxon has not been recorded spitting venom, despite having fangs modified for this behaviour [14]. This may be a result of the very small number of recorded interactions with humans.

### Distribution and habitat

The distribution of *Hemachatus nyangensis* appears to be restricted to the immediate vicinity of the Nyanga National Park and Nyanga District in the Eastern Highlands of Zimbabwe. It has been recorded from montane grasslands with areas of miombo woodland (Fig 5). The Eastern Highlands run along the eastern edge of Zimbabwe and into Mozambique, forming part of southern Africa’s Great Escarpment. Containing numerous endemic animal and plant species [47], the Highlands are included within the Eastern Afromontane Biodiversity Hotspot, an area with high potential for undiscovered biodiversity [48]. It is possible that this species also occurs in neighbouring Mozambique, as both the habitat and climatic conditions are similar.

## Discussion

### Molecular dating and biogeography

Our dated phylogeny of *Hemachatus* and related elapids corresponds largely (except for the interrelationships of the subgenera of *Naja*) to the tree of Kazandjian et al. [13]. Despite very obvious regional differences in colour pattern [49, 50], the rinkhals populations across southern Africa displayed little mitochondrial divergence, with diversification within *H*. *haemachatus* beginning around 0.69 Mya. Conversely, we estimate that the *H*. *haemachatus* clade from southern Africa and its sister lineage, *H*. *nyangensis* from Zimbabwe, diverged approximately seven to 14 million years ago. *Hemachatus* are temperate adapted [51]. Following the mid-Miocene Climatic Optimum 17–15 Mya, global temperatures cooled significantly [52]. During this period of cooling the Antarctic ice-sheet was re-established by 10 Mya, and the Arctic ice-sheet formed for the first time by 3.2 Mya [52]. On this cooling Earth, southern Africa was becoming more arid, due in part to cold water flowing northward along the western shore of southern Africa after the opening of the Drake Passage between South America and Antarctica [53]. The increasing aridity of lowland areas is likely to have caused significant range changes and fragmentation, particularly in temperate adapted species [54, 55]. Indeed, a dated phylogeny for *Strongylopus* frogs suggests a split between *S*. *rhodesianus* in the Eastern Highlands and the ancestor of *S*. *fasciatus* and *S*. *merumontanus* approximately 16 Mya [54]. Similar vicariance can be observed in *Bradypodion* chameleons in the mid to late Miocene [55], and the tortoise species *Psammobates tentorius* [56]). The same fragmentation likely affected the ancestors of *H*. *haemachatus* and *H*. *nyangensis*. Areas of suitable habitat across southern Africa shrank as it became drier, with lowland areas becoming subtropical, splitting *Hemachatus* between the temperate Eastern Highlands and the southern temperate biogeographic zone. Indeed, the Nyanga Massif where *H*. *nyangensis* is found is a close-knit bundle of mountains containing Zimbabwe’s highest point, Mount Nyangani, and most of the Massif is over 2000 metres above sea level. As a result of this high elevation, the Nyanga Massif has a cooler and wetter climate than the surrounding lowlands. At least two other temperate-adapted snake species, the viperid *Bitis atropos* and the lamprophiiid *Amplorhinus multimaculatus*, display a similarly fragmented distribution as *Hemachatus*, with isolated populations in the Eastern Highlands; both warrant further systematic and phylogeographic attention.

### Implications for the evolution of spitting in elapid snakes

Based on molecular dating of the initial divergence of spitting cobras in Africa (subgenus *Afronaja*) and Asia (spitting clade of the subgenus *Naja*), Kazandjian et al. [13] proposed that this defensive adaptation may have arisen as a result of the evolution of early hominins in Africa and their later arrival in Asia. *Hemachatus* was uninformative with respect to this hypothesis, as the origin of spitting was unconstrained along the long branch leading to a monotypic *Hemachatus*, offering little insight into when spitting emerged in this group. The discovery of a highly divergent new species in the genus allows the minimum age of the timing of the evolution of spitting in this lineage to be re-evaluated. Our point estimate of 10.14 Mya for the divergence of Zimbabwean and southern African *Hemachatus* significantly predates the estimated divergence between the lineages leading to *Pan* and *Homo* at 7.65 Mya [57], but our 95% HPD of 6.25–14.39 Mya encompasses the point estimate and the entire 95% credibility interval of that study (6.73–8.76 Mya).The reliance on short fragments of *12S* and *16S* rDNA sequence necessitated by poor preservation of the Zimbabwean rinkhals sample, and the resulting broad maximum credibility intervals preclude robust inferences on the timing of the focal node and any association with potential selective drivers for spitting. However, the apparent great age of the *H*. *haemachatus*—*H*. *nyangensis* divergence suggests that this taxon could be of considerable interest in the investigation of the potential drivers and correlates of the evolution of spitting. If *H*. *nyangensis* is extant, fresh material would provide the data required to more rigorously test the hypothesis of an association between hominin origins and the evolution of spitting in this third spitting elapid lineage.

### Museum DNA, species descriptions, and the future of the Nyanga rinkhals

We have provided proof of concept that, with appropriate museum genomic methods, even extremely low-quality museum samples can yield enough DNA sequence to infer the status of an undescribed species. Here, it has allowed us to identify and describe a cryptic and potentially extinct species of snake. The Eastern Highlands are under pressure from habitat modification by agriculture, illegal logging, and small-scale gold mining activities, and as a result of these factors the area has changed dramatically since the 1980s [48]. Invasive plant species are also modifying the montane grassland of the Nyanga Massif [47]. While *H*. *nyangensis* has not been seen since 1988, Broadley and Blaylock [16] suggest it should be searched for in remaining unaltered habitat in the eastern parts of Nyanga National Park. In recent years there have been a few notable cases of species which were thought to be extinct being rediscovered [58, 59], including Jackson’s climbing salamander (*Bolitoglossa jacksoni*), Wallace’s giant bee (*Megachile pluto*), and Voeltzkow’s chameleon (*Furcifer voeltzkowi*). The description of *H*. *nyangensis* sp. nov. draws attention to the importance of Zimbabwe’s Eastern Highlands as a region of high biodiversity and endemism. We remain hopeful that the species will eventually be rediscovered in the less anthropised parts of the Nyanga Highlands and strongly encourage renewed fieldwork to confirm its existence. Much of the area is Nyanga National Park, incorporating an area of 472 km^2^ and encompassing half of the Nyanga Massif, hopefully affording the species some protection if it is extant. Understanding the ecological requirements of this species (climate, diet, habitat structure) is essential for an assessment of its conservation status and the formulation of a recovery plan. Additional genetic sampling and sequencing will help resolve the status and origin of this mysterious snake, and contribute to our understanding of the biogeographic significance of the Eastern Highlands of Zimbabwe. It will also help to clarify the ecological context of the origin of venom spitting, and the role our ancestors might have played in driving this defensive innovation.

## Supporting information

S1 File Supplementary tables. PCR programs, GenBank accession numbers, and morphological data.
(DOCX)
