# Supplementary material for: Museum DNA reveals a new, potentially extinct species of rinkhals (Serpentes: Elapidae: Hemachatus) from the Eastern Highlands of Zimbabwe
Source: PLoS One. 2023 Sep 27;18(9):e0291432. doi: 10.1371/journal.pone.0291432 (PMC10529548; doi:10.1371/journal.pone.0291432)
Supplement: S1 File — PCR programs, GenBank accession numbers, and morphological data. (DOCX) [file pone.0291432.s001.docx]

**Supplementary material for “Museum DNA reveals a new, potentially extinct species of rinkhals (Serpentes: Elapidae: *Hemachatus*) from the Eastern Highlands of Zimbabwe”.**

**Tom Major, Pia Renk, Jens Reissig, Johanna L. A. Paijmans, Ellie Morris, Michael Hofreiter, Axel Barlow, Donald G. Broadley, Wolfgang Wüster**

**S1 Table.** PCR program for 12S (Goebel et al., 1999)

| Step | Thermocycler | Temperature (°C) | Time |
| --- | --- | --- | --- |
| 1 | Initial denature | 96 | 2 mins |
| 2 | Annealing | 52 | 45 secs |
| 3 | Extension | 72 | 2 mins |
| 4 | Denaturation | 94 | 30 secs |
| 5 | Annealing | 52 | 45 secs |
| 6 | Extension | 72 | 90 secs |
|  | Repeat 4-6 x 39 |  |  |
| 7 | Extension | 72 | 10 mins |
| 8 | Cooling | 4 | 10 mins |

**S2 Table.** PCR program for 16S (Palumbi, 1996)

| Step | Thermocycler | Temperature (°C) | Time |
| --- | --- | --- | --- |
| 1 | Initial denature | 94 | 2 mins |
| 2 | Denature | 94 | 30 secs |
| 3 | Annealing | 50 | 30 secs |
| 4 | Extension | 72 | 45 |
|  | Repeat 2 - 4 x 35 |  |  |
| 5 | Final extension | 72 | 5 mins |
| 6 | Cooling | 4 | 10 mins |

**S3 Table.** Morphological data used in the *Hemachatus* morphometric analysis.

| 2413 | 2415 | AMNH51838 | AMNH51839 | BM.11.7.1a | BM.45.2.21.104 | BM.55.10.16 | BM.55.10.16.303 | BM.55.10.16.303 | BM.62.9.2.26 | NMSR1963 | NMSR3892 | NMZB17611 | NMZB5553 | NMZB7066 | NMZB979 | NMZB-UM19762 | NMZB-UM2067 | UM12027 | UM5860 | UM9631 | UM9632 | NMSR5119 | NMSR5120 | NMSR5121 | NMZB9503 | Paget.Coll. | UM11847 | UM11848 | UM1307 | UM13413 | UM16098 | UM23767 | UM3061 | UM31317 | UM31402 | Museum_number |
| --- | --- | --- | --- | --- | --- | --- | --- | --- | --- | --- | --- | --- | --- | --- | --- | --- | --- | --- | --- | --- | --- | --- | --- | --- | --- | --- | --- | --- | --- | --- | --- | --- | --- | --- | --- | --- |
| Uilenkraal, Gansbaai, On K and J Groenewald Farm | Uilenkraal, Gansbaai, On K and J Groenewald Farm | Kroonstad | Big Tugela Valley | Cape of Good Hope | South Africa | South Africa | South Africa | South Africa | Cape of Good Hope | Johannesburg | Port Elizabeth | Standord, near Hermanus, Western Cape Province | ? | Johannesburg | Eastern Cape Province | South Africa' | Polela | Port Elizabeth | Port Elizabeth | Port Elizabeth | Lothair | Nyamaziwa, Inyanga National Park | Nyamaziwa, Inyanga National Park | Inyanga National Park | Trout Hatchery, Inyanga National Park | Charles Hanmer Park, Troutbeck, Inyanga | Mare Dah, Inyanga National Park | Mare Dah, Inyanga National Park | 5 km NW of Pungwe View, Inyanga National Park | Manyoli, Inyanga | Inyanga National Park | Marika, Inyanga | Mare Dah, Inyanga National Park | Charles Hanmer Park, Troutbeck, Inyanga | Troutbeck, Inyanga | Locality |
| Western Cape | Western Cape | Orange Free State | Natal | Cape Province | Cape Province | Cape Province | Cape Province | Cape Province | Cape Province | Transvaal | Cape Province | Cape Province | Cape Province | Transvaal | Cape Province | Orange Free State | Natal | Cape Province | Cape Province | Cape Province | Transvaal | Zimbabwe | Zimbabwe | Zimbabwe | Zimbabwe | Zimbabwe | Zimbabwe | Zimbabwe | Zimbabwe | Zimbabwe | Zimbabwe | Zimbabwe | Zimbabwe | Zimbabwe | Zimbabwe | State |
| other | other | other | other | other | other | other | other | other | other | other | other | other | other | other | other | other | other | other | other | other | other | zim | zim | zim | zim | zim | zim | zim | zim | zim | zim | zim | zim | zim | zim | Group |
| 688 | 328 | 940 | NA | NA | NA | NA | NA | NA | NA | NA | NA | 590 | 750 | 1080 | 1000 | 155 | 170 | NA | NA | NA | 650 | NA | NA | NA | 625 | 615 | 270 | 140 | 635 | 206 | 865 | 560 | 578 | 142 | 490 | SVL |
| 142 | 76 | 200 | NA | NA | NA | NA | NA | NA | NA | NA | NA | 130 | 165 | 185 | 210 | 31 | 26 | NA | NA | NA | 150 | NA | NA | NA | 121 | 127 | 55 | 22 | 133 | 39 | 150 | 125 | 102 | 26 | 97 | Tail_length |
| 830 | 404 | 1140 | NA | NA | NA | NA | NA | NA | NA | NA | NA | 720 | 915 | 1265 | 1210 | 186 | 196 | NA | NA | NA | 800 | NA | NA | NA | 746 | 742 | 325 | 162 | 768 | 245 | 1015 | 685 | 680 | 168 | 587 | Total_length |
| 2 | 1 | 1 | 1 | 1 | 2 | 1 | 1 | 1 | 2 | 2 | 1 | 1 | 1 | 2 | 1 | NA | NA | 1 | 1 | 2 | 1 | NA | NA | NA | 2 | 1 | 1 | 2 | 1 | 2 | 2 | 1 | 2 | 1 | 2 | Sex |
| NA | NA | 17 | 19 | NA | NA | NA | NA | NA | NA | 17 | 19 | 19 | 19 | 17 | 19 | 17 | 19 | 17 | 17 | 17 | 17 | NA | NA | NA | 17 | 17 | 17 | 17 | 18 | 17 | 17 | 17 | 17 | 16 | 17 | Nape_scale_rows |
| NA | NA | 19 | 19 | NA | NA | NA | NA | NA | NA | 19 | 19 | 19 | 19 | 17 | 19 | 18 | 19 | 19 | 19 | 19 | 17 | 17 | NA | 17 | 17 | 17 | 18 | 19 | 19 | 19 | 19 | 19 | 17 | 17 | 17 | Midbody_scale_rows |
| NA | NA | 13 | 13 | NA | NA | NA | NA | NA | NA | 13 | 14 | 13 | 13 | 13 | 13 | 13 | 13 | 13 | 13 | 13 | 13 | NA | NA | NA | 13 | 13 | 13 | 13 | 13 | 13 | 13 | 13 | 13 | 13 | 11 | Pre-cloacal_scale_rows |
| NA | NA | 134 | 138 | 120 | 129 | 117 | 118 | 121 | 140 | 145 | 134 | 127 | 135 | 143 | 131 | NA | NA | 134 | 133 | 148 | 130 | NA | NA | NA | 130 | 122 | 122 | 126 | 123 | 130 | 128 | 124 | 126 | 119 | 128 | Ventrals |
| NA | NA | E | E | NA | NA | NA | NA | NA | NA | E | E | E | E | E | E | E | E | E | E | E | E | NA | NA | NA | E | E | E | E | E | E | E | E | E | E | E | Cloacal |
| 39 | 39 | 46 | 44 | 39 | 35 | 37 | 38 | 35 | 39 | 43 | 43 | 42 | 42 | 38 | 43 | 43 | 37 | 44 | 38 | 40 | 41 | NA | NA | NA | 34 | 37 | 35 | 30 | 38 | 37 | 34 | 38 | 35 | 34 | 33 | Sub-caudals |
| NA | NA | 7 | 7 | NA | NA | NA | NA | NA | NA | 7 | 7 | 7 | 7 | 7 | 7 | 6 | 7 | 7 | 7 | 7 | 7 | 7 | 7 | 7 | 7 | 7 | 6.5 | 7 | 7 | 7 | 7 | 7 | 7 | 7 | 7 | Supralabials |
| NA | NA | 3,4 | 3,4 | NA | NA | NA | NA | NA | NA | 3,4 | 3,4 | 3,4 | 3,4 | 3,4 | 3,4 | 3 | 3,4 | 3,4 | 3,4 | 3,4 | 3,4 | 3,4 | 3,4 | 3,4 | 3,4 | 3,4 | 3,4 | 3,4 | 3,4 | 3,4 | 3,4 | 3,4 | 3/3,4 | 3,4 | 3,4 | Upper_labials_enter_orbit |
| NA | NA | NA | NA | NA | NA | NA | NA | NA | NA | 8 | 8 | 8 | 8 | 7.5 | 8 | 8 | 8 | 8.5 | 8 | 9 | 8 | 8 | 8 | 8 | 8 | 7.5 | 8 | 8 | 8 | 8 | 8 | 8 | 8 | 8 | 8 | Lower_labials |
| NA | NA | NA | NA | NA | NA | NA | NA | NA | NA | 4 | 4 | 3.5 | 4 | 4 | 4 | 4 | 4 | 4 | 4 | 4 | 4 | 4 | 4 | 4 | 4 | 3.5 | 4 | 4 | 4 | 4 | 4 | 4 | 4 | 4 | 4 | In_contact_ant |
| NA | NA | 1 | 1 | NA | NA | NA | NA | NA | NA | 1 | 1 | 1 | 1 | 1 | 1 | 1 | 1 | 1 | 1 | 1 | 1 | 1 | 1 | 1 | 1 | 1 | 1 | 1 | 1 | 1 | 1 | 1 | 1 | 1 | 1 | Preoculars |
| NA | NA | 3 | 3 | NA | NA | NA | NA | NA | NA | 3 | 3 | 3 | 3 | 3 | 3 | 3 | 3 | 3 | 3 | 3 | 3 | 3 | 3 | 3 | 3 | 3 | 3 | 3 | 3 | 3 | 3 | 3 | 3 | 3 | 3 | Postoculars |
| NA | NA | 2+3 | 2+3 | NA | NA | NA | NA | NA | NA | 2+3 | 2+3/2+5 | 2+3 | 2+3 | 2+3 | 2+3 | 2+3 | 2+3 | 2+3 | 2+3 | 2+4/2+3 | 2+3 | 2+2/2+3 | 2+3 | 2+3 | 2+3 | 2+3 | 2+3 | 2+3 | 2+2/2+3 | 2+2/2+3 | 2+3 | 2+3 | 2+3 | 2+3 | 2+3/2+2 | Temporals |
| 19.47 | 19.47 | 27.2 | 30 | 18.4 | NA | NA | NA | NA | NA | NA | 25 | 19 | NA | NA | NA | NA | 29.7 | 25 | 25 | 25 | 30.4 | 32 | 32 | 32 | 32 | 32 | 32 | 32 | 32 | 32 | 32 | 32 | 32 | 32 | 32 | x |
| -34.56 | -34.56 | -27.6 | -28 | -34.3 | NA | NA | NA | NA | NA | NA | -33 | -34 | NA | NA | NA | NA | -29.8 | -33 | -33 | -33 | -26.3 | 18 | 18 | 18 | 18 | 18 | 18 | 18 | 18 | 18 | 18 | 18 | 18 | 18 | 18 | y |

**S4 Table.** Samples and GenBank accession numbers for specimens used in the genetic analysis.

| Species | Description | 12S | 16S | Cytochrome b | NADH 4 |
| --- | --- | --- | --- | --- | --- |
| *Aspidelaps scutatus* | - | [U96790](https://www.ncbi.nlm.nih.gov/nucleotide/U96790.1?report=genbank&log$=nucltop&blast_rank=1&RID=KKKYENE2013) | [KX694617](https://www.ncbi.nlm.nih.gov/nucleotide/KX694617.1?report=genbank&log$=nucltop&blast_rank=1&RID=KKKZAJBV013) | [AF217828](https://www.ncbi.nlm.nih.gov/nucleotide/AF217828.1?report=genbank&log$=nucltop&blast_rank=1&RID=KKKTD3MS013) | [AY058969](https://www.ncbi.nlm.nih.gov/nucleotide/AY058969.1?report=genbank&log$=nucltop&blast_rank=3&RID=KKKV9M4S013) |
| ***Hemachatus inyangae*** **sp. nov.** | NMZB 9503 Nyanga, Zimbabwe | OQ857311 | OQ857301 | - | - |
| *Hemachatus haemachatus* | 2337 KwaZulu-Natal | OQ857312 | OQ857302 | MT346639 | MT346831 |
| *Hemachatus haemachatus* | 2658 KwaZulu-Natal | OQ857313 | OQ857303 | - | - |
| *Hemachatus haemachatus* | 2263 Eswatini | OQ857314 | OQ857304 | MT346634 | MT346826 |
| *Hemachatus haemachatus* | 2361 Western Cape | OQ857315 | OQ857305 | MT346641 | MT346833 |
| *Hemachatus haemachatus* | 2596 Eastern Cape | OQ857316 | OQ857306 | - | - |
| *Hemachatus haemachatus* | 2338 KwaZulu-Natal | OQ857317 | OQ857307 | MT346640 | MT346832 |
| *Hemachatus haemachatus* | 2320 Gauteng | OQ857318 | OQ857308 | MT346638 | MT346830 |
| *Naja nigricollis* | 1074 | EU624237 | [GQ359754](https://www.ncbi.nlm.nih.gov/nucleotide/GQ359754.1?report=genbank&log$=nucltop&blast_rank=1&RID=KKM9T2X9013) | GQ359505 | AY713377 |
| *Naja ashei* | 1430  Watamu, Kenya | GQ359656 | GQ359742 | GQ359493 | GQ359575 |
| *Naja mossambica* | 190 (12S, cytb), 1391 (ND4) | GQ359658 | [GQ359744](https://www.ncbi.nlm.nih.gov/nucleotide/GQ359744.1?report=genbank&log$=nucltop&blast_rank=1&RID=KKK212KR013) | [MT346654](https://www.ncbi.nlm.nih.gov/nucleotide/MT346654.1?report=genbank&log$=nucltop&blast_rank=1&RID=KKH1EGK601R) | [MT346851](https://www.ncbi.nlm.nih.gov/nucleotide/MT346851.1?report=genbank&log$=nucltop&blast_rank=1&RID=KKJVG05Z016) |
| *Naja katiensis* | 1540  Doussoudiana, Mali | GQ359657 | GQ359743 | GQ359494 | GQ359576 |
| *Naja pallida* | 1080  Tanzania | GQ359659 | GQ359745 | [GQ359496](https://www.ncbi.nlm.nih.gov/nucleotide/GQ359496.1?report=genbank&log$=nucltop&blast_rank=1&RID=KKK7EEX9013) | [GQ359578](https://www.ncbi.nlm.nih.gov/nucleotide/GQ359578.1?report=genbank&log$=nucltop&blast_rank=2&RID=KKK9NAGW013) |
| *Naja nubiae* | 837 (12S, 16S) 4565 (cytb, ND4) | GQ359660 | GQ359746 | [MT346686](https://www.ncbi.nlm.nih.gov/nucleotide/MT346686.1?report=genbank&log$=nucltop&blast_rank=1&RID=KKKD75PG016) | [MT346881](https://www.ncbi.nlm.nih.gov/nucleotide/MT346881.1?report=genbank&log$=nucltop&blast_rank=1&RID=KKKE07B1013) |
| *Naja nivea* | 1295 | EU624238 | GQ359755 | MT346759 | MT346935 |
| *Naja arabica* | 1681 | GQ359663 | GQ359749 | [GQ359500](https://www.ncbi.nlm.nih.gov/nucleotide/GQ359500.1?report=genbank&log$=nucltop&blast_rank=5&RID=KKMGB3ZR013) | [GQ387077](https://www.ncbi.nlm.nih.gov/nucleotide/GQ387077.1?report=genbank&log$=nucltop&blast_rank=1&RID=KKMHGCUX016) |
| *Naja haje* | Egypt 893 | GQ359664 | GQ359750 | [GQ359501](https://www.ncbi.nlm.nih.gov/nucleotide/GQ359501.1?report=genbank&log$=nucltop&blast_rank=1&RID=KKMP7R5C013) | [GQ387063](https://www.ncbi.nlm.nih.gov/nucleotide/GQ387063.1?report=genbank&log$=nucltop&blast_rank=1&RID=KKMPX5V1013) |
| *Naja haje* | Kenya 1262 | GQ359661 | GQ359747 | GQ359498 | GQ359580 |
| *Naja senegalensis* | Senegal 2203 (cytb, ND4), Mali 1542 (12S, 16s) | [GQ359666.](https://www.ncbi.nlm.nih.gov/nucleotide/GQ359666.1?report=genbank&log$=nucltop&blast_rank=1&RID=KKN5SD0A013) | [GQ359752](https://www.ncbi.nlm.nih.gov/nucleotide/GQ359752.1?report=genbank&log$=nucltop&blast_rank=1&RID=KKN2TYD1013) | [MT346763.](https://www.ncbi.nlm.nih.gov/nucleotide/MT346763.1?report=genbank&log$=nucltop&blast_rank=1&RID=KKMXXB9V013) | GQ387080 |
| *Naja annulifera* | 881 | GQ359667 | GQ359753 | [GQ387119](https://www.ncbi.nlm.nih.gov/nucleotide/GQ387119.1?report=genbank&log$=nucltop&blast_rank=2&RID=KKNUKCE101R) | [GQ387090](https://www.ncbi.nlm.nih.gov/nucleotide/GQ387090.1?report=genbank&log$=nucltop&blast_rank=1&RID=KKNVRJDY01R) |
| *Naja annulata* | - | [U96792](https://www.ncbi.nlm.nih.gov/nucleotide/U96792.1?report=genbank&log$=nucltop&blast_rank=1&RID=KKNYCFRH01R) | [AY188049](https://www.ncbi.nlm.nih.gov/nucleotide/AY188049.1?report=genbank&log$=nucltop&blast_rank=1&RID=KKNZFABW01R) | [MT346699](https://www.ncbi.nlm.nih.gov/nucleotide/MT346699.1?report=genbank&log$=nucltop&blast_rank=1&RID=KKP17Y8X01R) | [MT346895](https://www.ncbi.nlm.nih.gov/nucleotide/MT346895.1?report=genbank&log$=nucltop&blast_rank=1&RID=KKP2WG9C013) |
| *Naja naja* | ABTC32465 | EU547088 | EU547137 | EU547039 | EU546997 |
| *Naja naja* | 579 | [EU624236](https://www.ncbi.nlm.nih.gov/nucleotide/EU624236.1?report=genbank&log$=nucltop&blast_rank=1&RID=KKPANW6U013) | [GQ359756](https://www.ncbi.nlm.nih.gov/nucleotide/GQ359756.1?report=genbank&log$=nucltop&blast_rank=3&RID=KKPB7D3T01R) | [MT346711](https://www.ncbi.nlm.nih.gov/nucleotide/MT346711.1?report=genbank&log$=nucltop&blast_rank=1&RID=KKP9F7DE013) | [MT346907](https://www.ncbi.nlm.nih.gov/nucleotide/MT346907.1?report=genbank&log$=nucltop&blast_rank=1&RID=KKPA043U013) |
| *Naja atra* | 582 (Hong Kong - cytb, ND4), mitogenome (12S, 16S) | [EU921898](https://www.ncbi.nlm.nih.gov/nucleotide/EU921898.1?report=genbank&log$=nucltop&blast_rank=1&RID=KKPGSNSE01R) | [EU921898](https://www.ncbi.nlm.nih.gov/nucleotide/EU921898.1?report=genbank&log$=nucltop&blast_rank=1&RID=KKPHGDPC01R) | [MT346703](https://www.ncbi.nlm.nih.gov/nucleotide/MT346703.1?report=genbank&log$=nucltop&blast_rank=9&RID=KKPFDVD5013) | [MT346899](https://www.ncbi.nlm.nih.gov/nucleotide/MT346899.1?report=genbank&log$=nucltop&blast_rank=1&RID=KKPG680W01R) |
| *Naja kaouthia* | 812  Southern Vietnam | [LC431744](https://www.ncbi.nlm.nih.gov/nucleotide/LC431744.1?report=genbank&log$=nucltop&blast_rank=1&RID=KKPR1307013) | [LC431744](https://www.ncbi.nlm.nih.gov/nucleotide/LC431744.1?report=genbank&log$=nucltop&blast_rank=1&RID=KKPR1307013) | [LC431744](https://www.ncbi.nlm.nih.gov/nucleotide/LC431744.1?report=genbank&log$=nucltop&blast_rank=1&RID=KKPR1307013) | [LC431744](https://www.ncbi.nlm.nih.gov/nucleotide/LC431744.1?report=genbank&log$=nucltop&blast_rank=1&RID=KKPR1307013) |
| *Naja sputatrix* | 584  Java, Indonesia | OQ857319 | OQ857309 | MT346730 | MT346922 |
| *Naja sumatrana* | 586 (ND4, cytb) | [JN687928](https://www.ncbi.nlm.nih.gov/nucleotide/JN687928.1?report=genbank&log$=nucltop&blast_rank=1&RID=KKS3GZ2A013) | [JN687929](https://www.ncbi.nlm.nih.gov/nucleotide/JN687929.1?report=genbank&log$=nucltop&blast_rank=1&RID=KKS2VUKC013) | [MT346735](https://www.ncbi.nlm.nih.gov/nucleotide/MT346735.1?report=genbank&log$=nucltop&blast_rank=1&RID=KKS4W366013) | [MT346931](https://www.ncbi.nlm.nih.gov/nucleotide/MT346931.1?report=genbank&log$=nucltop&blast_rank=2&RID=KKS49SMN013) |
| *Naja siamensis* | - | [JN687926](https://www.ncbi.nlm.nih.gov/nucleotide/JN687926.1?report=genbank&log$=nucltop&blast_rank=1&RID=KKSAB6P9013) | [JN687927](https://www.ncbi.nlm.nih.gov/nucleotide/JN687927.1?report=genbank&log$=nucltop&blast_rank=1&RID=KKSAUBGR013) | [MT346726](https://www.ncbi.nlm.nih.gov/nucleotide/MT346726.1?report=genbank&log$=nucltop&blast_rank=1&RID=KKS97S5W013) | [MT346924](https://www.ncbi.nlm.nih.gov/nucleotide/MT346924.1?report=genbank&log$=nucltop&blast_rank=1&RID=KKS9XS44013) |
| *Ophiophagus hannah* | China | EU921899 | EU921899 | EU921899 | EU921899 |
| *Ophiophagus hannah* | Indonesia | AZIM01009253 | AZIM01009253 | AZIM01009253 | AZIM01009253 |
| *Pseudohaje goldii* | 1336 | OQ857320 | OQ857310 | MT346778 | MT346952 |
| *Walterinnesia aegyptia* | - | [U96807](https://www.ncbi.nlm.nih.gov/nucleotide/U96807.1?report=genbank&log$=nucltop&blast_rank=1&RID=KKSKUU8Y013) | [HQ267785](https://www.ncbi.nlm.nih.gov/nucleotide/HQ267785.1?report=genbank&log$=nucltop&blast_rank=1&RID=KKSMAUJ0016) | [MT346780](https://www.ncbi.nlm.nih.gov/nucleotide/MT346780.1?report=genbank&log$=nucltop&blast_rank=2&RID=KKSKEKK2013) | [AY058988](https://www.ncbi.nlm.nih.gov/nucleotide/AY058988.1?report=genbank&log$=nucltop&blast_rank=1&RID=KKSPSD2N01R) |

References

Goebel AM, Donnelly JM, Atz ME. PCR Primers and Amplification Methods for 12S Ribosomal DNA, the Control Region, Cytochrome Oxidase I, and Cytochrome b in Bufonids and Other Frogs, and an Overview of PCR Primers which Have Amplified DNA in Amphibians Successfully. Molecular Phylogenetics and Evolution. 1999;11: 163–199. doi:<https://doi.org/10.1006/mpev.1998.0538>

Palumbi SR. Nucleic Acids II: The Polymerase Chain Reaction. In: Hillis DM, Moritz C, Mable BK, editors. Molecular Systematics. Sinauer, Sunderland; 1996. pp. 205–247.
